# Biochemical and Structural Insights into the Mechanisms of SARS Coronavirus RNA Ribose 2′-O-Methylation by nsp16/nsp10 Protein Complex

**DOI:** 10.1371/journal.ppat.1002294

**Published:** 2011-10-13

**Authors:** Yu Chen, Ceyang Su, Min Ke, Xu Jin, Lirong Xu, Zhou Zhang, Andong Wu, Ying Sun, Zhouning Yang, Po Tien, Tero Ahola, Yi Liang, Xinqi Liu, Deyin Guo

**Affiliations:** 1 State Key Laboratory of Virology and Modern Virology Research Center, College of Life Sciences, Wuhan University, Wuhan, P. R. China; 2 Institute of Biotechnology, University of Helsinki, Helsinki, Finland; 3 College of Life Sciences, Nankai University, Tianjin, P. R. China; 4 Institute of Medical Virology, Wuhan University School of Medicine, Wuhan, P. R. China; Purdue University, United States of America

## Abstract

The 5′-cap structure is a distinct feature of eukaryotic mRNAs, and eukaryotic viruses generally modify the 5′-end of viral RNAs to mimic cellular mRNA structure, which is important for RNA stability, protein translation and viral immune escape. SARS coronavirus (SARS-CoV) encodes two S-adenosyl-L-methionine (SAM)-dependent methyltransferases (MTase) which sequentially methylate the RNA cap at guanosine-N7 and ribose 2′-O positions, catalyzed by nsp14 N7-MTase and nsp16 2′-O-MTase, respectively. A unique feature for SARS-CoV is that nsp16 requires non-structural protein nsp10 as a stimulatory factor to execute its MTase activity. Here we report the biochemical characterization of SARS-CoV 2′-O-MTase and the crystal structure of nsp16/nsp10 complex bound with methyl donor SAM. We found that SARS-CoV nsp16 MTase methylated m7GpppA-RNA but not m7GpppG-RNA, which is in contrast with nsp14 MTase that functions in a sequence-independent manner. We demonstrated that nsp10 is required for nsp16 to bind both m7GpppA-RNA substrate and SAM cofactor. Structural analysis revealed that nsp16 possesses the canonical scaffold of MTase and associates with nsp10 at 1∶1 ratio. The structure of the nsp16/nsp10 interaction interface shows that nsp10 may stabilize the SAM-binding pocket and extend the substrate RNA-binding groove of nsp16, consistent with the findings in biochemical assays. These results suggest that nsp16/nsp10 interface may represent a better drug target than the viral MTase active site for developing highly specific anti-coronavirus drugs.

## Author Summary

The distinctive feature of eukaryotic mRNAs is the presence of methylated cap structure that is required for mRNA stability and protein translation. As all viruses employ cellular ribosomes for protein translation, most cytoplasmically replicating eukaryotic viruses including coronaviruses have evolved strategies to cap their RNAs. It was shown very recently that ribose 2′-O-methylation in the cap structure of viral RNAs plays an important role in viral escape from innate immune recognition. The 2′-O-methyltransferase (2′-O-MTase) encoded by SARS coronavirus is composed of two subunits, the catalytic subunit nsp16 and the stimulatory subunit nsp10, which is different from all other known 2′-O-MTases that are partner-independent. Here we show that the role of nsp10 is to promote nsp16 to bind capped RNA substrate and the methyl donor S-adenosyl-L-methionine (SAM). We solved the crystal structure of the nsp16/nsp10/SAM complex, and the structural analysis revealed that the details of the inter-molecular interactions and indicated that nsp10 may stabilize the SAM-binding pocket and extend the capped RNA-binding groove. The interaction interface of nsp16/nsp10 is unique for coronaviruses and thus may provide an attractive target for developing specific antiviral drugs for control of coronaviruses including the deadly SARS coronavirus.

## Introduction

Coronaviruses are etiological agents of respiratory and enteric diseases in livestock, companion animals and humans, exemplified by severe acute respiratory syndrome coronavirus (SARS-CoV) which was responsible for a worldwide SARS outbreak in 2003 and caused over 8000 cases of infection with about 10% fatality rate. They are characterized by possessing the largest and most complex positive-stranded RNA genome (ranging from 27 to 31 kb) among RNA viruses. Fourteen open reading frames (ORFs) have been identified in the genome of SARS-CoV, of which 12 are located in the 3′-one third of the genome, encoding the structural and accessory proteins translated through a nested set of subgenomic RNAs [1], [2]. The 5′-proximal two thirds of the genome comprise 2 large overlapping ORFs (1a and 1b), which encode two large replicase polyproteins that are translated directly from the genome RNA, with 1b as the frameshifted extension of 1a. These two precursor polyproteins are cleaved into 16 mature replicase proteins, named as non-structural protein (nsp) 1–16, which form the replication-transcription complex (RTC) localized in endoplasmic reticulum-derived membranes [3], [4]. Strikingly, the coronavirus genome is predicted to encode several RNA processing enzymes that are not common to small RNA viruses [1], including nsp14 as an exoribonuclease and guanine N7-methyltransferase (N7-MTase) [5], [6], [7], [8] and nsp15 as a nidovirus-specific endonuclease [9], [10].

Eukaryotic and most viral mRNAs possess a 5′-terminal cap structure, in which an N7-methyl-guanine moiety is linked to the first transcribed nucleotide by a 5′-5′ triphosphate bridge [11], [12]. The cap structure is essential for efficient splicing, nuclear export, translation and stability of eukaryotic mRNA [13], [14], [15], [16]. All viruses use the translational machinery of host cells. With the exception of some viruses, such as picornaviruses and hepatitis C virus that circumvent the capping problem by using an internal ribosome entry site (IRES) for mRNA translation [17], [18], viruses of eukaryotes have evolved diversified strategies to cap their mRNAs that are thus translated by cap-dependent mechanisms in the manner of eukaryotic mRNAs. It has been suggested for three decades that coronavirus mRNA may carry a 5′-cap structure [19], [20], [21], [22], but the principal enzymes involved in coronavirus RNA capping and their biochemical mechanisms have not been characterized until recently. Cap formation of eukaryotic and viral mRNAs requires universally three sequential enzymatic reactions. First, an RNA triphosphatase (TPase) removes the γ-phosphate group from the 5′-triphosphate end (pppN) of the nascent mRNA chain to generate the diphosphate 5′ -ppN. Subsequently, a RNA guanylyltransferase (GTase) transfers a GMP to the 5′-diphosphate end to yield the cap core structure (GpppN). Then a N7-MTase methylates the capping guanylate at the N7 position to produce a cap-0 structure (m7GpppN) [13]. While lower eukaryotes, including yeast, employ a cap-0 structure, higher eukaryotes and viruses usually further methylate the cap-0 structure at the ribose 2′-O position of the first and second nucleotide of the mRNA by a ribose 2′-O MTase to form cap-1 and cap-2 structure, respectively [13]. Very recently, it was shown that ribose 2′-O-methylation of viral RNA cap provides a mechanism for viruses to escape host immune recognition [23], [24].

For coronaviruses, several nsps have been indicated to be involved in viral RNA capping. We have shown that SARS-CoV nsp14, a previously described exoribonuclease, acts as N7-MTase to generate cap-0 structure [8]. Recently, it was shown that SARS-CoV nsp16 acts as 2′-O-MTase in complex with nsp10 and selectively 2′-O-methylates the cap-0 structure to give rise to cap-1 structure [25]. *Feline coronavirus* (FCoV) nsp16 was also shown to bind N7-methyl guanosine cap (m^7^GpppAC_3-6_) and methylate the penultimate nucleotide at 2′-O position to yield a cap-1 structure in vitro [26]. Coronavirus nsp13 has been shown to exhibit RNA TPase activity *in vitro* and thus proposed to be functional in RNA capping reaction [27], [28], but a direct role for nsp13 in RNA capping still awaits experimental evidence. Currently, the RNA GTase that is essential for cap formation is completely unknown for coronaviruses.

SARS-CoV nsp16 requires nsp10 as a stimulatory factor to execute its 2′-O-MTase activity [25] and this also holds true for other coronaviruses (our unpublished results). This mechanism is unique for 2′-O-MTase of coronaviruses and has not been found in any other viruses or host cells. However, the molecular mechanisms underlying the enzymatic activity of nsp16 and the stimulatory effect of nsp10 are unknown. Here we report the crystal structure of the heterodimer of nsp16 and nsp10 (nsp16/nsp10) with bound methyl donor SAM and biochemical characterization of the stimulation mechanisms. We found that nsp10 is required for nsp16 to bind both SAM and RNA substrate, and the crystal structure shows that nsp10 may stabilize the SAM-binding pocket and extend the RNA-binding groove of nsp16. These results have implications for designing specific anti-coronavirus drugs to control infection.

## Results

### Specific 2′-O-methylation of GpppA-capped RNA by SARS-CoV nsp14/nsp16/nsp10 complex

**Figure 1:**
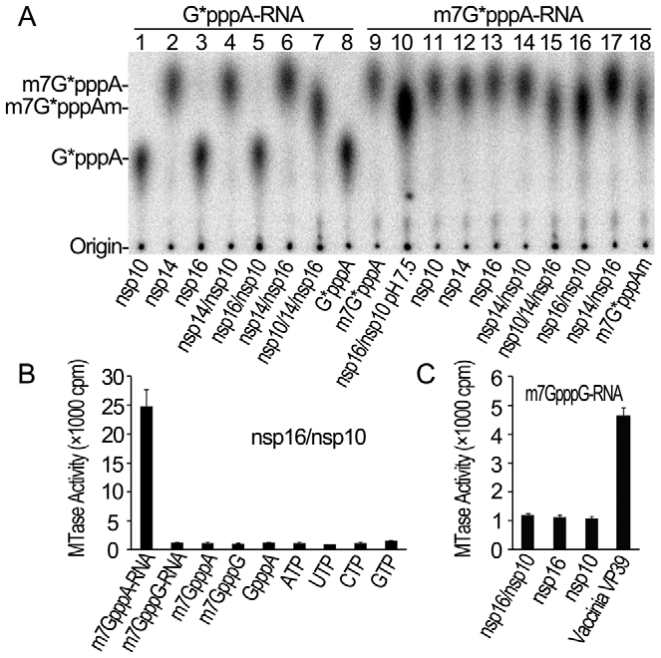
Biochemical analyses of the MTase activities of SARS-CoV nsp14, nsp16 and nsp10. (A) TLC analysis of nuclease P1-resistant cap structures released from ^32^P-labeled G*pppA-RNA methylated by nsp10, nsp14, nsp16, nsp14/nsp10, nsp16/nsp10, nsp14/nsp16, and nsp10/nsp14/nsp16, respectively (lanes 1–7), and m7G*pppA-RNA methylated by nsp16/nsp10 (at pH 7.5), nsp10, nsp14, nsp16, nsp14/nsp10, nsp10/nsp14/nsp16, nsp16/nsp10, and nsp14/nsp16, respectively (at pH 8.0) (lanes 10–17). The markers G*pppA (lane 8), m7G*pppA (lane 9), and m7G*pppAm (lane 18) were prepared with commercial vaccinia virus capping enzymes. The positions of origin and migration of G*pppA, m7G*pppA, and m7G*pppAm (lanes 8, 9, and 18) are indicated on the left. The bands located between origin and G*pppA are free α-^32^P-GTP, which may be left over after one-step purification of labeled RNA. (B) Different RNA substrates were used to test the methylation activities of nsp16/nsp10 complex of SARS-CoV (*n* = 3, mean values ± SD). (C) ^32^P-labeled G*pppG-RNA was used as substrate to test the methylation activities of nsp16/nsp10, nsp16, and nsp10 respectively. Vaccinia VP39 2′-O-MTase was used as a positive control (*n* = 3, mean values ± SD).

In our previous work, we adopted a genetic screening system and biochemical assays to identify SARS-CoV nsp14 as N7-MTase [8]. However, we did not observe any 2′-O-MTase activity in various biochemical assays for SARS-CoV nsp16, which was previously predicted to be 2′-O-MTase [1], [29], although a low 2′-O-MTase activity was demonstrated for feline coronavirus nsp16 [26]. We and others showed previously that SARS-CoV nsp10 could interact with both nsp14 and nsp16 [30], [31], suggesting a role for nsp10 in the functions of nsp14 and nsp16. Therefore, we undertook to test the effects of nsp10 on the MTase activities of both nsp14 and nsp16. As shown in Figure 1A, by using radiolabeled and unmethylated G*pppA-capped RNA as substrate (where the * indicates that the following phosphate was ^32^P labeled, and the sequence is identical with viral genomic RNA except for the second nucleotide), nsp14 alone could efficiently N7-methylate GpppA-RNA to generate cap-0 structure (m7GpppA) (Figure 1A, lane 2) and nsp10 did not significantly alter nsp14 N7-MTase activity in our testing system (Figure 1A, lane 4). While either nsp16 or nsp10 alone did not show any MTase activity (Figure 1A, lanes 3 and 1), the mixture of nsp10/nsp14/nsp16 gave rise to cap-1 structure (m7GpppAm) (Figure 1A, lane 7), indicating that addition of nsp10 and nsp16 rendered the complex active in the 2′-O-MTase reaction. We then used radiolabeled m7G*pppA-capped RNA substrate and demonstrated that nsp14 could not 2′-O-methylate RNA cap-0 structure (Figure 1A, lane 12) but the mixture of nsp16 and nsp10 (nsp16/nsp10) did possess the 2′-O-MTase activity to convert cap-0 to cap-1 structure at pH 7.5 and 8.0 (Figure 1A, lanes 10 and 16). These results indicate that nsp10 may function as a stimulatory factor for nsp16 and is required for the 2′-O-MTase activity of nsp16. While this work was ongoing, similar observations were made by Bouvet et al. [25].

Our previous studies showed that SARS-CoV nsp14 N7-MTase could N7-methylate both GpppA- and GpppG- capped RNA in a sequence-independent manner [8]. As both the genomic and subgenomic RNAs of SARS-CoV all start with an adenine, we tested whether SARS-CoV nsp16 2′-O-MTase has sequence specificity by using m7GpppA- and m7GpppG-capped RNAs as substrates. As shown in Figures 1B and 1C, SARS-CoV nsp16/nsp10 complex gave a strong methylation signal on m7GpppA-capped RNA substrate but not on m7GpppG-capped RNA, suggesting that SARS-CoV nsp16/nsp10 functions in cap sequence-specific manner. The VP39 2′-O-MTase of vaccinia virus was used as a control in the methylation assay system (Figure 1C). Furthermore, we observed that nsp16/nsp10 could not methylate cap analogues and individual NTPs (Figure 1B), indicating that the RNA substrate for nsp16/nsp10 needs to contain a stretch of nucleotides linked to the cap. A recent study showed that the cap with a 5 nucleotide extension was sufficient as substrate for nsp16 [25]. In this short RNA substrate, only the first nucleotide is identical with the first nucleotide A in the SARS-CoV genome RNA and the remaining 4 nucleotides are different from the genomic sequence. Taken together, these results indicate that the first nucleotide A is the sequence determinant for nsp16 methylation specificity. These results also suggest that nsp16 or nsp16/nsp10 complex need to form an RNA-binding groove that has enough space and affinity to accommodate m7GpppA-capped RNA with an extension of a few of nucleotides. Binding of the cofactor SAM and substrate RNA to MTase is the prerequisite for its enzymatic activity, and therefore we next tested whether SARS-CoV nsp10 might promote the SAM- and RNA-binding capability of nsp16.

### Nsp10 promotes the binding of the cofactor SAM to nsp16

**Figure 2:**
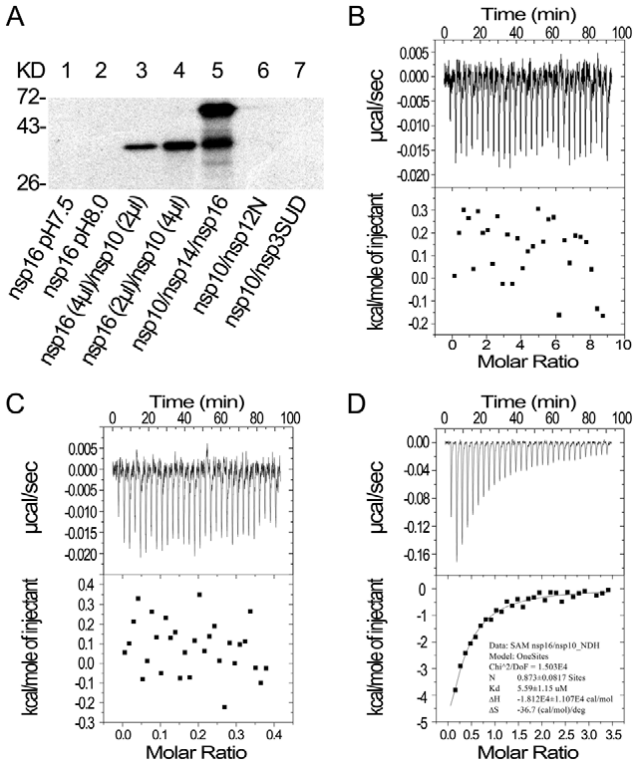
SAM binding analyses of SARS-CoV nsp14, nsp16 and nsp10. (A) 12% SDS-PAGE analysis of SAM UV cross-linking with nsp16 (at pH 7.5 and 8.0) (lanes 1–2), nsp16/nsp10 with different ratios (lanes 3–4), and nsp10/nsp14/nsp16 (lane 5). Nsp10/nsp12N and nsp10/nsp3-SUD (lanes 6–7) were used as controls. The positions of marker proteins are indicated on the left. (B to D) ITC profiles for the binding of SAM to nsp16 (B), nsp10 (C), and nsp16/nsp10 complex (D), respectively. The top panels represent the raw data for sequential injections of SAM (150 µM) into nsp16, nsp10 and nsp16/nsp10 complex. The bottom panels show the plots of the heat evolved (kilocalories) per mole of SAM.

SAM is the methyl donor for both N7- and 2′-O-MTase in RNA cap methylation, and high affinity binding and correct positioning of the cofactor in the SAM-binding site of the MTase provides the basis for the methyl transfer into the substrate RNA. Therefore, we first tested whether nsp10 could influence the binding affinity of nsp16 toward SAM. As shown in Figure 2A, nsp16 alone or the complex of nsp10 and control proteins (nsp12N and nsp3-SUD) were not able to bind SAM at different pH values (Figure 2A, lanes 1, 2, 6, and 7). However, nsp16 could bind SAM when complexed with nsp10 (Figure 2A, lanes 3 and 4). In the mixture nsp10/nsp14/nsp16, both nsp14 and nsp16 could bind to SAM (Figure 2A, lane 5). There were no signals at the position of nsp10 in SAM binding assays (data not shown). These results demonstrate that nsp10 could boost nsp16 to bind the methyl donor SAM.

To further study the different SAM binding affinity of SARS-CoV nsp16/nsp10 complex and nsp16 or nsp10 alone, isothermal titration calorimetry (ITC) was used to measure the thermodynamic changes during SAM-binding. ITC profiles for the binding of SAM to nsp16, nsp10 and nsp16/nsp10 complex (Figures 2B, 2C and 2D, respectively) of SARS-CoV showed that the complex of SARS-CoV nsp16 and nsp10 could bind SAM specifically but either nsp16 or nsp10 alone could not. The top panels in Figures 2B-D show raw ITC curves resulting from the injections of SAM into a solution of nsp16 (Figure 2B), nsp10 (Figure 2C), and nsp16/nsp10 complex of SARS-CoV (Figure 2D). The titration curves show that SAM binding to nsp16/nsp10 complex is exothermic, resulting in negative peaks in the plots of power versus time (Figure 2D). However, adding SAM to either nsp16 or nsp10 alone is apyretic, resulting in random peaks around 0-baseline in the plots of power versus time (Figures 2B and 2C). The bottom panels in Figures 2B-D plot the heat evolved per mole of SAM added, corrected for the heat of SAM dilution, against the molar ratio of SAM to nsp16 (Figure 2B), nsp10 (Figure 2C), and nsp16/nsp10 complex of SARS-CoV (Figure 2D). The thermodynamic parameters for the binding between SAM and nsp16/nsp10 complex (*N* = 0.873±0.0817 sites, 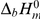 = −18.12±11.07 kcal mol^−1^, *K*_d_ = 5.59±1.15 µM, and 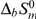 = −36.7 cal mol^−1^ K^−1^) were obtained by fitting the data to a single set of identical sites model, indicating that SAM bound to nsp16/nsp10 complex with a moderate affinity in the absence of DTT. The *N* value indicated the binding stoichiometry and it suggested that the molecular ratio of SAM to nsp16/nsp10 complex is 1∶1. As the observed N value is less than 1, it may indicate that not all nsp16 bound with nsp10 to form nsp16/nsp10 complex in the assays. As shown by the value of dissociation constant (*K*_d_), the binding affinity between SAM and nsp16/nsp10 complex was moderate. The mutual binding affinity of SARS-CoV nsp10 and nsp16 was also measured by ITC, and the *K*_d_ value was 2.11±0.97 µM, which indicated similar binding affinity to that of SAM and nsp16/nsp10 complex. Taken together, these results showed that nsp10 is required for nsp16 to bind the methyl donor SAM and the binding affinity of SAM and nsp16/nsp10 complex is moderate.

### Nsp10 assists nsp16 in binding m7GpppA-capped RNA

**Figure 3:**
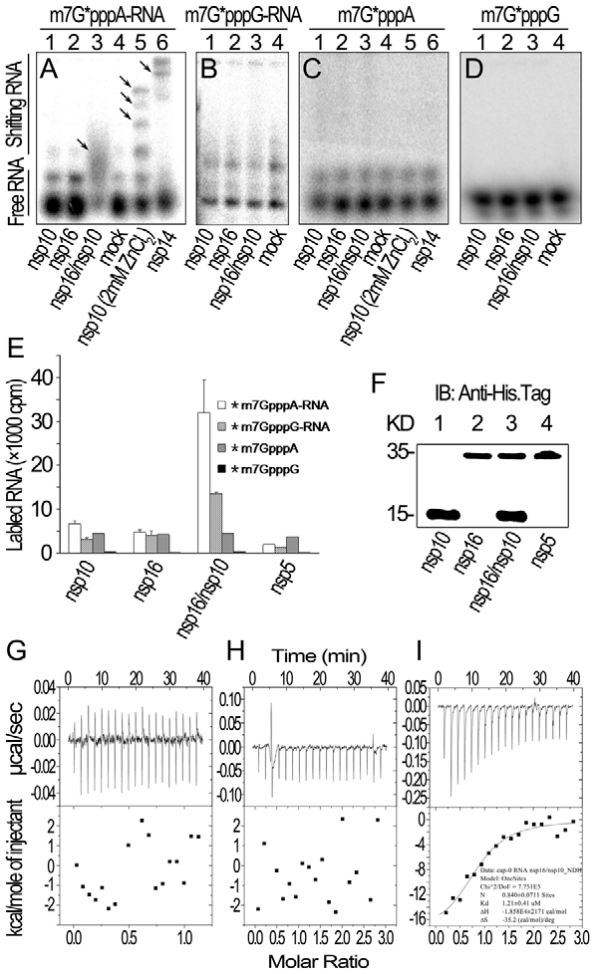
RNA substrate binding analyses of nsp10 and nsp16 of SARS-CoV. (A) Gel shift assays were performed by 8% N-PAGE to analyze the binding of ^32^P-labeled m7G*pppA-RNA incubated with nsp10, nsp16, and nsp16/nsp10, respectively (lanes 1–3). There was no protein in the mock as negative control (lane 4). Nsp10 (with 2 mM ZnCl_2_) and nsp14 were used as positive controls (lanes 5–6). (B) ^32^P-labeled m7G*pppG-RNA was incubated with nsp10, nsp16, nsp16/nsp10, and mock respectively (lanes 1–4). Mixtures were analyzed by 8% N-PAGE. (C) ^32^P-labeled m7G*pppA cap analogue was incubated with different proteins as in (A). Mixtures were analyzed by 14% N-PAGE. (D) ^32^P-labeled m7G*pppG cap analogue was incubated with different proteins as in (B). Mixtures were analyzed by 14% N-PAGE. Positions of the free RNA substrates and shifting RNA substrates are indicated on the left. Black arrows indicate shifting RNA bands in each lane. (E) Different ^3^H-labeled RNA substrates were used to test the binding affinities to nsp10, nsp16, and nsp16/nsp10. His_6_-nsp5 was a negative control (*n* = 2, mean values ± SD). (F) 30 µL of the final suspensions from (E) were analyzed by Western Blotting analysis. (G to I) ITC profiles for the binding of nsp16 (G), nsp10 (H), and nsp16/nsp10 complex (I), respectively to m7GpppA-capped RNA. The top panels represent the raw data for sequential injections of nsp16, nsp10 and nsp16/nsp10 complex into m7GpppA-capped RNA (7 µM). The bottom panels show the plots of the heat evolved (kilocalories) per mole of purified proteins.

We then tested whether nsp10 is required for specific binding of m7GpppA-capped RNA. In gel shift assays in the absence of zinc ions, neither nsp10 nor nsp16 could shift the RNA bands (Figure 3A, lanes 1 and 2) but nsp14 and the complex of nsp16 and nsp10 could (Figure 3A, lanes 6 and 3). In the presence of zinc ions, nsp10 as zinc finger protein could bind RNA non-specifically (Figure 3A, lane 5). The complex of nsp16 and nsp10 did not associate with m7GpppG-capped RNA (Figure 3B) and cap analogues (m7GpppA and m7GpppG) (Figures 3C and 3D). These results indicate that SARS-CoV nsp10 promotes nsp16 to specifically bind m7GpppA-capped RNA. To confirm these results, we adopted pull-down assays by using hexahistidine-tagged proteins and radiolabeled RNAs or cap analogues. As shown in Figure 3E, pull-down of nsp16 and nsp10 mixture by nickel-nitrilotriacetic acid (Ni-NTA) resin gave rise to high level of radioactive signal for *m7GpppA-capped RNA (where * indicates that the methyl group was ^3^H labeled) while nsp10, nsp16, or nsp5 alone could not pull down the labeled RNA. Also in this testing system, nsp16/nsp10 complex had a low binding affinity to *m7GpppG-capped RNA and cap analogues (*m7GpppA and *m7GpppG) (Figure 3E). The pulled-down proteins were further checked by Western blotting to confirm the presence of the indicated proteins (Figure 3F). These results collectively showed that the nsp16 could bind specifically to m7GpppA-capped RNA only in the presence of nsp10.

Furthermore, we also analyzed the binding specificity and thermodynamics between nsp16/nsp10 and capped RNA in ITC assays. The thermodynamic changes are shown for nsp16 (Figure 3G), nsp10 (Figure 3H) and nsp16/nsp10 complex of SARS-CoV (Figure 3I) when added to the solution of m7GpppA-capped RNA, respectively. The top panels in Figures 3G-I show raw ITC curves and the bottom panels plot the heat evolved per mole of the injected protein, corrected for the heat of the corresponding proteins dilution, against the molar ratio to m7GpppA-capped RNA. The titration curves show that m7GpppA-capped RNA binding to nsp16/nsp10 complex is exothermic, resulting in negative peaks in the plots of power versus time (Figure 3I). However, all others were apyretic, resulting in random peaks around 0-baseline in the plots of power versus time (Figures 3G and 3H). The binding affinity of nsp16/nsp10 complex with m7GpppA-capped RNA is higher than that with SAM as shown by the thermodynamic parameters for the binding between nsp16/nsp10 complex and m7GpppA-capped RNA (*N* = 0.840±0.0711 sites, 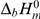 = −18.58±2.171 kcal mol^−1^, *K*_d_ = 1.21±0.41 µM, and 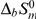 = −35.2 cal mol^−1^ K^−1^). We further analysed the thermodynamic changes when cap analogues (m7GpppA and m7GpppG) were added to nsp16/nsp10 complex in ITC, but neither of the two cap analogues showed exothermic binding (data not shown), which suggested that the cap analogues are not the substrate of nsp16/nsp10 complex.

All together, these data showed that nsp10 plays an essential role in the specific binding of m7GpppA-capped RNA by nsp16. However, m7GpppG-capped RNA and cap analogues can not bind to the nsp16/nsp10 complex, and thus can not be used as substrate by 2′-O-MTase of SARS-CoV.

Based on the results presented above, it is suggested that SARS-CoV nsp10 may either stabilize the SAM-binding pocket of nsp16 or change the conformation of nsp16 so as to efficiently take in and hold the SAM molecule. Furthermore, the association of nsp10 with nsp16 may provide a proper groove for binding and holding of m7GpppA-capped RNA substrate. We thus expected to reveal the details of SAM- and RNA-binding and stimulation mechanisms from the crystal structure of nsp16/nsp10.

### Structure determination and general features of the nsp16/nsp10 complex

To obtain crystals of nsp16/nsp10 protein complex combined with its MTase co-substrate SAM, 6×histidine-tagged nsp16 and 6×histidine-tagged nsp10 with nsp11 extension were expressed individually in *Escherichia coli* cells and co-purified with Ni-NTA resin. The protein mixture of purified nsp16 and nsp10 was supplemented with methyl donor SAM to obtain protein complex and then applied to crystallization by the hanging-drop vapor diffusion method. Crystals appeared readily in hanging drops and were diffracted to high resolution at 2.0 Å under X-rays from synchrotron radiation source. The structure was solved subsequently by multi-wavelength anomalous diffraction method taking advantage of selenomethionine substituted crystals (see Materials and Methods). Within one asymmetric unit in the crystals, one nsp10, one nsp16 and one SAM molecule were identified unambiguously. Residues in both nsp10 and nsp16 were clearly traced except the C-terminus after Ser129 in nsp10 and the nsp11 extension. The atomic coordinates of the structure have been deposited in the Protein Data Bank (PDB) as entry 3R24.

**Figure 4:**
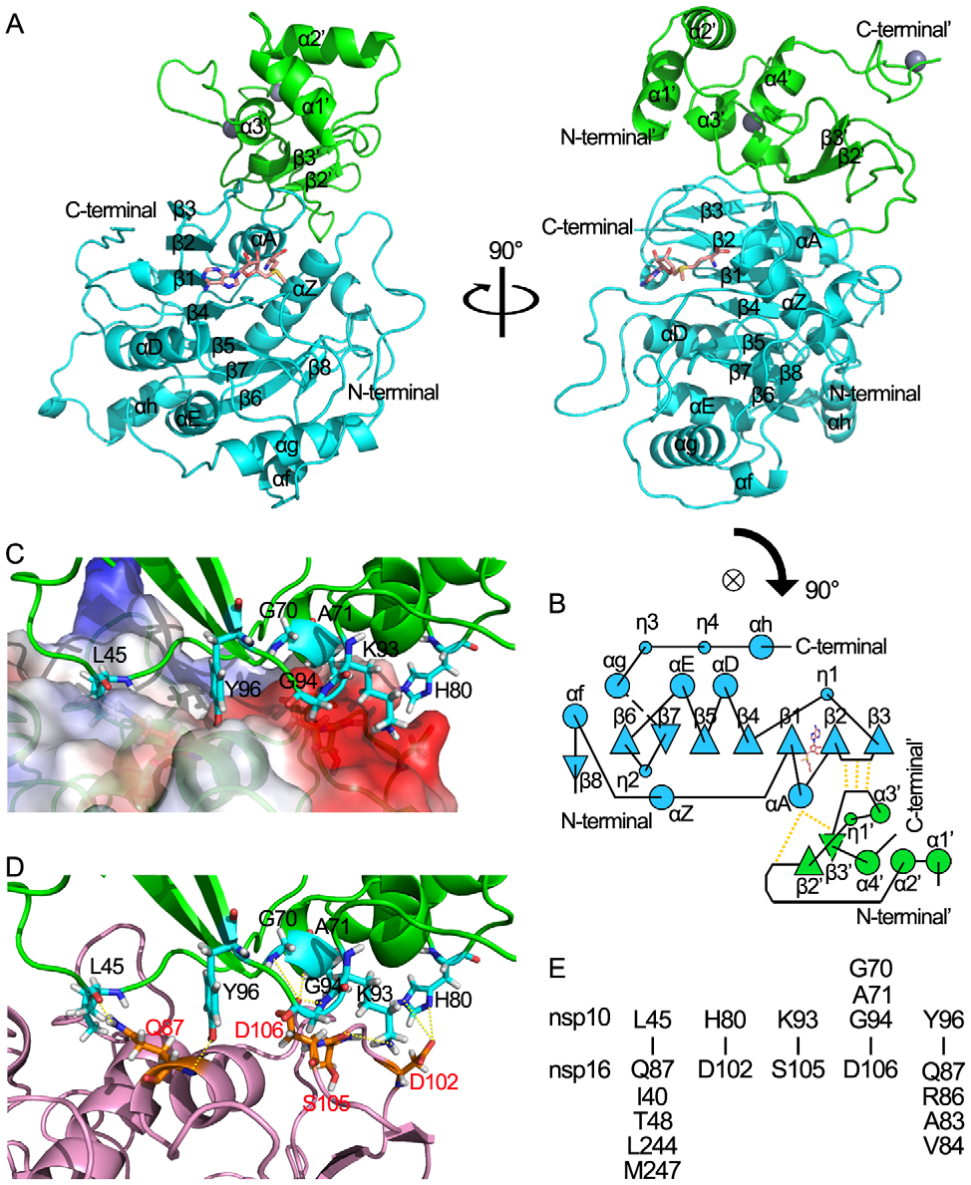
Structural insight into the nsp16/nsp10 complex of SARS-CoV. (A) Two orthogonal views of the overall structure of nsp16/nsp10 complex. The proteins are shown in ribbon with green (nsp10) and cyan (nsp16). SAM is depicted in a stick model and colored by atoms (C: salmon, O: red, N: blue, H: white). (B) Schematic diagram of topology of the nsp16/nsp10 complex colored by proteins as in (A). Hydrogen bonds located in the interaction sites are shown by dotted lines. (C) Interaction surface of nsp16/nsp10 complex. The main chain of nsp10 is show in ribbon with green. Residues (Leu-45, Gly-70, Ala-71, His-80, Lys-93, Gly-94, Tyr-96) which interact with nsp16 are depicted in a stick model and colored by atoms (C: cyan, O: red, N: blue, H: white). Nsp16 is shown in ribbon with electrostatic potential surface of 20% transparency. The surface charge is depicted as blue in positively charged areas, red in negatively charged areas, and white in electroneutral areas. (D) Interaction surface of nsp16/nsp10 complex. The main chain of nsp10 is show as ribbon and colored in green. Residues (Leu-45, Gly-70, Ala-71, His-80, Lys-93, Gly-94, Tyr-96) which interact with nsp16 are shown as sticks and colored by atoms (C: cyan, O: red, N: blue, H: white). The main chain of nsp16 is show as ribbon and colored in light magenta. Residues (Gln-87, Asp-102, Ser-105, Asp106) which interact with nsp10 are shown as sticks and colored by atoms (C: orange, O: red, N: blue, H: white). Hydrogen bonds and salt bridges are shown as yellow dotted lines. (E). Residues involved in the direct interaction of nsp16 and nsp10 are listed in the lower and upper parts, respectively.

The structure of nsp16 (Figures 4A and 4B) exhibits the characteristic fold of the class I MTase family, comprising a seven-stranded β-sheet surrounded by α-helices and loops [32]. Search of the PDB using DALI [33] identified high structural similarity of nsp16 with FtsJ (PDB entry 1EIZ), a partner-independent MTase from *E. coli* [34], with a 2.6 Å root-mean square deviation (RMSD) for 179 aligned Cα (from Y30 to A209 of nsp16 with 16% sequence identity and Dali Z-score 26.6). The residues 30-209 of nsp16 form the core MTase domain. Nevertheless, some differences between the two structures are evident. The most significant difference lies in the αD helix, which is visible on the surface of FtsJ (Figure S1E) but invisible for nsp16 (Figure S1B). The αD helix is important for both SAM-binding and RNA cap-binding, and it is very short in partner-dependent nsp16 2′-O-MTase but relatively long in all other known viral 2′-O-MTases, including vaccinia virus VP39 (PDB entry 1AV6) (Figure S1C), Dengue virus NS5 MTase (PDB entry 1L9K) (Figure S1D), and Bluetongue virus VP4 2′-O-MTase (PDB entry 2JHP) (Figure S1F), which are partner-independent MTases. The differences between nsp16 and other 2′-O-MTases are shown in the structure-based alignment of 2′-O-MTases (vaccinia virus VP39, Flavivirus NS5 MTase, and FtsJ) (Figure S2).

The structure of the stimulatory protein nsp10 in nsp16/nsp10 complex (Figures 4A and 4B) is consistent with the structure of nsp10 reported previously [35], [36],indicating that the structure of nsp10 is not impacted by the interaction between nsp16 and nsp10. Nsp10 can be roughly segregated into three regions: a helical domain at the N terminus followed by an irregular β-sheet region, and a C-terminal loop region. Two zinc ions were clearly identified in nsp10, which formed the center of two zinc fingers, one coordinated by Cys-74, Cys-77, His-83 and Cys-90, and the other coordinated by Cys-117, Cys-120, Cys-128 and Cys-130. The two zinc fingers render nsp10 the ability to bind polynucleotide chains in a nonselective manner in the presence of zinc ions [35], [36], [37].

During the review process of this work, a structure of nsp16/nsp10 complex of SARS-CoV was reported by Decroly and colleagues [38], which is generally the same as the structure described in this work. The major difference lies in that the structure we solved contains the methyl donor SAM that was purposely supplemented in the protein mixture, and the one by Decroly and colleagues contains S-adenosylhomocysteine (SAH) which is the product of SAM after methyltransfer and may be captured from the medium by nsp16 that was co-expressed with nsp10 in bacterial cells [38]. The atomic coordinates of nsp16/nsp10 structure solved by Decroly et al. have not been released until now, and thus detailed comparison of the two structures is not available.

### The nsp10/nsp16 interface

Nsp10 and nsp16 formed a protein complex through an interaction surface covering approximately 1767 Å^2^ in total, indicating a very stable interaction. The interaction surface on nsp10 was dominated by hydrophobic interactions in center with surrounding hydrophilic interactions. By using the online software Interfaces and Assemblies of EMBL-EBI (http://www.ebi.ac.uk/msd-srv/prot_int/pistart.html), it was found that significant contacts between nsp10 and nsp16 involve residues 40–47/69–84/93–96 of nsp10, and residues 37–48/76–91/102–110/244–248 of nsp16 (Figures 4). Close inspection revealed a cluster of important residues involved in nsp16-nsp10 interaction, including Val-42, Met-44, Gly-70, Ser-72, Arg-78, His-80, Lys-93, Gly-94, Lys-95 and Tyr-96 in nsp10, in agreement with hotspots identified in biochemical assays [39]. Intermolecular hydrogen bonds exist between Gly-70/Ala-71/Gly-94 of nsp10 and Asp-106 of nsp16, Lys-93 of nsp10 and Ser-105 of nsp16, Leu-45 of nsp10 and Gln-87 of nsp16, Tyr-96 of nsp10 and Ala-83/Gln-87 of nsp16. There are two salt bridges between His-80 (nitrogen atoms ND1 and NE2) of nsp10 and Asp-102 (oxygen atom OD2) of nsp16 (Figures 4 D and 4E). Hydrophobic interactions were involved between Leu-45 in nsp10 and a hydrophobic pocket composed of Ile-40/Thr-48/Leu-244/Met-247 in nsp16, and Tyr-96 in nsp10 and a hydrophobic pocket consisting of Val-84, main chain of Gln-87 and Arg-86 from nsp16 (Figures 4C and 4E).

**Figure 5:**
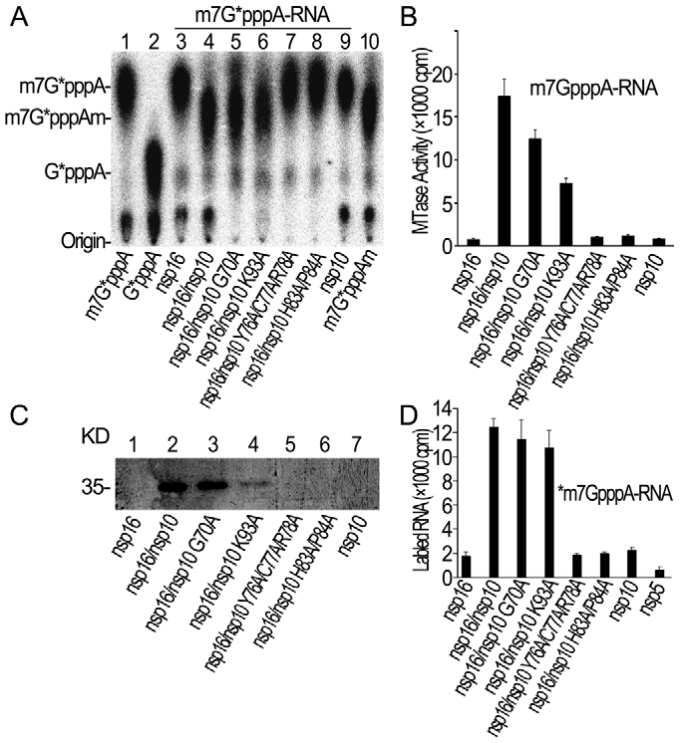
Biochemical analyses of nsp10 mutants. (A) TLC analysis of nuclease P1-resistant cap structures released from ^32^P-labeled m7G*pppA-RNA methylated by nsp16/nsp10 G70A, nsp16/nsp10 K93A, nsp16/nsp10 Y76A/C77A/R78A, and nsp16/nsp10 H83A/P84A (lanes 5–8), respectively. Nsp16, nsp16/nsp10, and nsp10 were used as controls (lanes 3, 4, and 9). The positions of origin and migration of m7G*pppA, G*pppA, and m7G*pppAm (lanes 1, 2, and 10) are indicated on the left. The bands located between origin and G*pppA are free α-^32^P-GTP. (B) m7GpppA-RNA was used to test the methylation activities of nsp10 mutants complexed with nsp16 of SARS-CoV (*n* = 2, mean values ± SD). (C) 12% SDS-PAGE analysis of SAM UV cross-linking with nsp16/nsp10 G70A, nsp16/nsp10 K93A, nsp16/nsp10 Y76A/C77A/R78A, and nsp16/nsp10 H83A/P84A (lanes 3–6), respectively. Nsp16, nsp16/nsp10, and nsp10 were used as controls (lanes 1, 2, and 7). (D) ^3^H-labeled *m7GpppA-RNA substrates were used to test the binding affinities to nsp16/nsp10 G70A, nsp16/nsp10 K93A, nsp16/nsp10 Y76A/C77A/R78A, and nsp16/nsp10 H83A/P84A. Nsp16, nsp10, and nsp5 were negative controls (*n* = 2, mean values ± SD).

Biochemical assays showed that only nsp16/nsp10 complex could bind the substrates m7GpppA-RNA and SAM, and execute the 2′-O MTase activity. Therefore, mutations on the interaction surface of nsp16/nsp10 complex which can block this interaction should influence the substrates binding, and consequently the MTase activity of nsp16. A double mutant (H83A/P84A) and a triple mutant (Y76A/C77A/R78A) at the interaction surface of nsp10 were generated. These mutants almost completely abolished the SAM (Figure 5C) and m7GpppA-RNA (Figure 5D) binding of nsp16, and also abrogated MTase activity (Figures 5A and 5B). The main chain N atom of Gly-70 in nsp10 formed a hydrogen bond with Asp-106 of nsp16. The single mutation G70A only slightly influences this main chain interaction, and accordingly, it slightly impaired the SAM- and RNA-binding activity of nsp16 (Figures 5C, lane 3 and 5D) and attenuated by 30% the 2′-O-MTase activity of nsp16 (Figures 5A, lane 5 and 5B). Similar results were obtained by Lugari et al., except for an Y96F mutation, which increased both the nsp10-nsp16 affinity and the MTase activity of nsp16 [39]. In the interaction surface of nsp16/nsp10, the side chain of Tyr-96 in nsp10 stacks to the hydrophobic pocket consisting of Val-84, main chain of Gln-87 and Arg-86 in nsp16. Compared with tryptophan, the side chain of phenylalanine exhibits a stronger hydrophobicity. Therefore, Y96F mutation may strengthen this hydrophobic interaction of nsp10 and nsp16, and thus enhance nsp16/nsp10 MTase activity. This observation suggests that the hydrophobic interaction by the aromatic nucleus of Tyr-96 is more important than the hydrogen bond made by its hydroxyl group for maintaining the interaction surface with nsp16. Taken together, biochemical analysis of the critical residues involved in the interaction interface was consistent with the structural observations.

### A unique mode of SAM binding to nsp16/nsp10 complex

**Figure 6:**
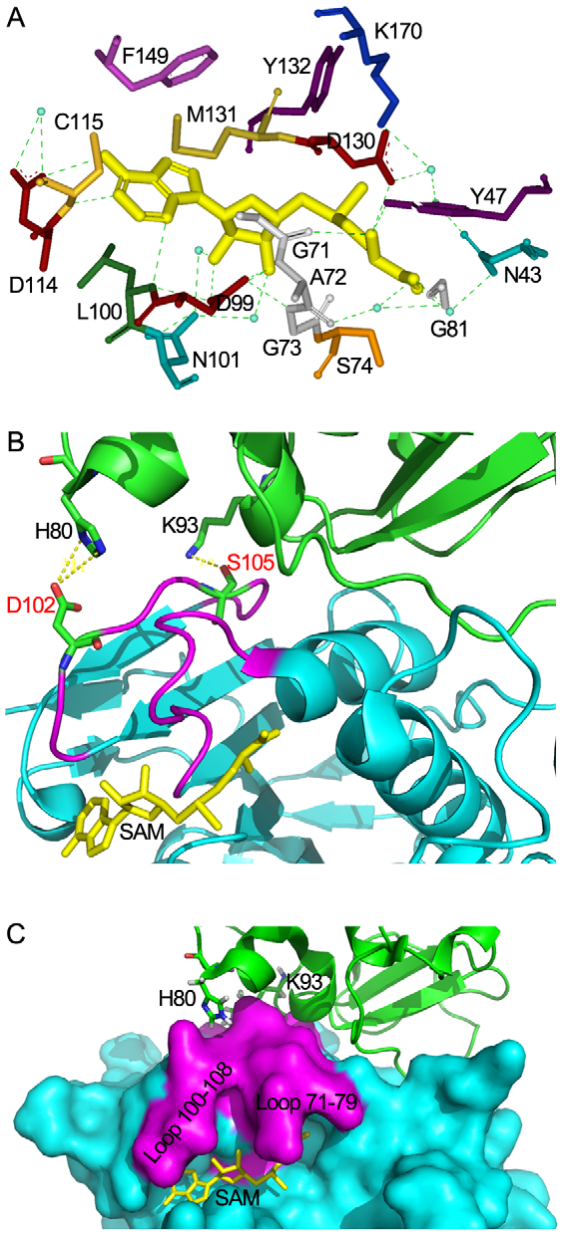
Structural mechanisms of nsp10 in stimulating the SAM binding of nsp16. (A) Residues of nsp16 interacting with SAM. All residues and SAM are shown as sticks, water as blue dots, and hydrogen bonds as green dotted lines. Nsp10-mediated stabilization of the SAM binding cleft of nsp16 in ribbon model (B) and surface model (C). Nsp16 is colored in cyan. Residues 71–79 and residues 100–108 are colored in magenta. Nsp10 is shown as ribbon and colored in green. SAM is colored in yellow. His-80 and Lys-93 are shown as sticks and colored by atoms (C: green, O: red, N: blue). Hydrogen bonds are shown as yellow dotted lines. In (B), Asp-102, Ser-105 of nsp16 are shown as sticks and colored by atoms (C: green, O: red, N: blue).

Methyl donor SAM was added to protein mixture used for crystal screening, and SAM molecule is visible in the crystal structure of nsp16/nsp10 complex. The ligand SAM lies at the C-terminus of strands β1 and β2, similar to most SAM-dependent MTases [32] (Figures 4A and 4B). As shown in Figure 6A, the adenosine moiety of SAM is stacked among Phe-149, Met-131 and Cys-115 in nsp16 through Van der Waals force, and polar contacts also exist, such as those of adenine N6 with side chain of Asp-114, N1 with main chain of Cys-115, and N3 with main chain of Leu-100. The 2′- and 3′-OH of the adenosine ribose of SAM are stabilized by the side chain of Asp-99 and the side chain of Asn-101 via hydrogen bonds. The hydrophobic patch of the ribose packs against Met-131 and the hydrophobic main chain of Tyr-132. The amino group of the methionine moiety of SAM is maintained by three polar contacts: N atom forms a hydrogen bond with main chain carbonyl group of Gly-71, side chain of Tyr-47 and Asp-130; hydroxyl O atom interacts with main chain of Gly-81 and main chain of Ala-72 and Gly-73; and carbonyl O atom interacts with side chain Asn-43 (Figure 6A).

The crystal structure of nsp16/nsp10 complex shows that the SAM binding cleft of nsp16 is composed of three loops: loop 71–79 (residues 71–79) together with loop 100–108 (residues 100–108) forming a wall on one side of the cleft and loop 130–148 (residues 130–148), which is followed by αD helix, forming a wall on the other side. Several ‘hot spot’ residues were located at the loop regions (Leu-100, Asn-101, Asp-130, Met-131, Tyr-132) (Figure S1A). Structural analysis revealed that the loop followed by αD helix of partner-independent 2′-O-MTases is shorter than that of nsp16 (loop 130–148) and is sustained by rigid αD helix such as to form a stable wall to keep the SAM inside the cleft. In contrast, in the SAM-binding pocket of nsp16, the corresponding loop (loop 130–148) is long and flexible while the supporting rigid αD helix is short (Figure S2). Moreover, the loop 100–108 of nsp16 is also longer and more flexible than that in other 2′-O-MTases. These structural features may make the SAM-binding cleft more flexible and thus in need of extra support from the stimulatory factor nsp10. In Flavivirus NS5 MTase, the loop corresponding to residues 100–108 was much longer for unknown reasons, but due to the strong sustaining effect of the long αD helix, the SAM-binding cleft of NS5 MTase is postulated to be more stable than that of nsp16. Therefore, it appears that at least one stable wall is essential for the SAM binding cleft to maintain the SAM binding activity. In the crystal structure of nsp16/nsp10 complex, one hydrogen bond forms between Lys-93 of nsp10 and Ser-105 of nsp16, and two salt bridges exist between His-80 ND1 and NE2 from nsp10 with Asp-102 OD2 from nsp16 (Figure 6B). Both Ser-105 and Asp-102 of nsp16 are located in the flexible loop region 100–108, which stabilizes one wall of the SAM binding cleft (Figures 6C and S1A), and consequently promotes the SAM binding activity of nsp16/nsp10 complex (Figure 2). This phenomenon of enhancing the SAM-binding activity by stabilization of the binding cleft via protein-protein interaction is observed for the first time, revealing a unique mode of SAM binding among the 2′-O-MTases.

### Structural insights into the RNA-binding activity of nsp16/nsp10 complex

**Figure 7:**
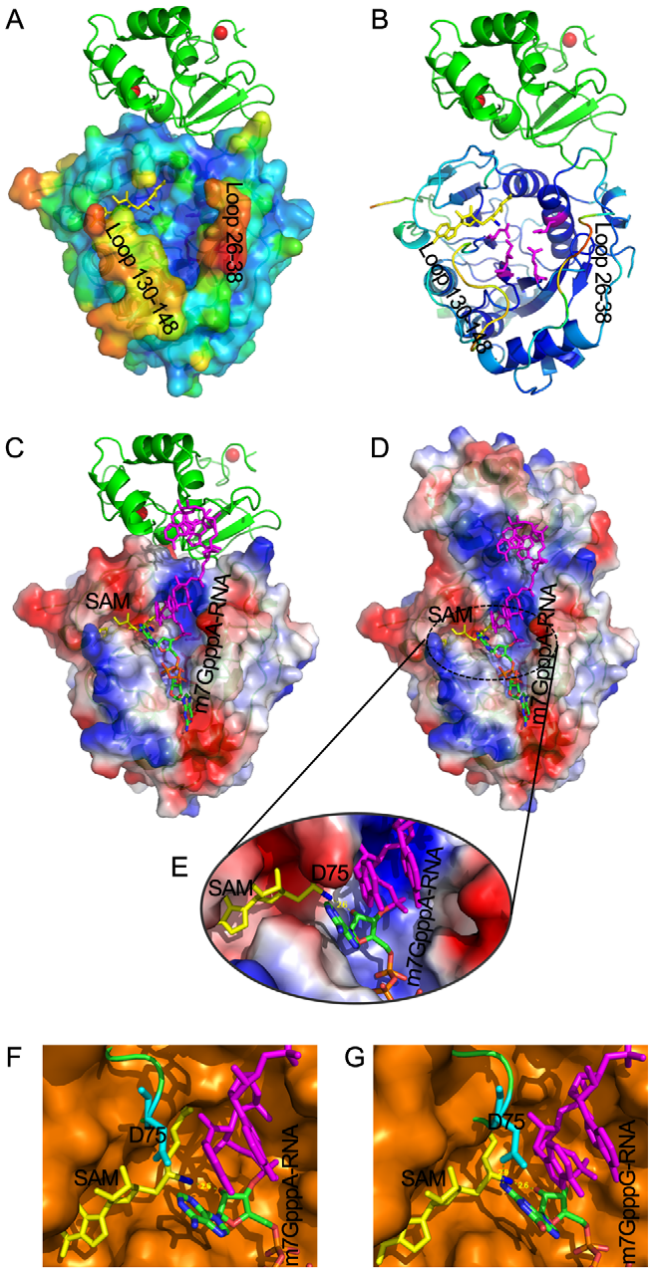
Structural mechanisms of nsp10 in stimulating the binding of capped RNA to nsp16. The cap-binding groove of nsp16 is shown as surface with 20% transparency (A) and ribbon (B). The structural model of nsp16/nsp10 complex is colored by the Debye-Waller factor, based on which the warm colored regions are highly flexible and mobile. The K-D-K-E motif residues of nsp16 (Lys-46, Asp-130, Lys-170 and Glu-203) are shown as sticks and colored in magenta. Nsp10 is shown as ribbon and colored in green. Molecular docking of m7GpppA-RNA or m7GpppG-RNA on RNA-binding groove of nsp16/nsp10 complex. Overall structure of docking model of nsp16/nsp10 complex and m7GpppA-RNA (C, D) and details of methyltransferase activity site of nsp16 (E). Nsp16 is shown as ribbon and electrostatic potential surface with 20% transparency. Nsp10 is shown as ribbon (C) or electrostatic potential surface with 20% transparency (D). SAM is shown as sticks and colored in yellow. The methyl group of SAM is colored in blue. Zinc ions are shown as spheres. The m7GpppA-RNA is shown as sticks and the first two nucleotides are colored by atoms (C: green, O: red, N: blue, P: orange) and the next nucleotides are colored in magenta. Structural alignment of methyltransferase activity site from VP39 (PDB entry: 1AV6) and SARS-CoV nsp16 docking with m7GpppA-RNA (F) or m7GpppG-RNA (G), respectively. VP39 is superimposed with SARS-CoV nsp16 (F, G). VP39 is shown as surface and colored in orange, and nsp16 is shown as ribbon and colored in green, with Asp-75 (D75) shown as sticks and colored in cyan.

Previous studies have demonstrated the existence of a conserved motif for methyl-transfer: K-D-K-E residues among various 2′-O MTases which catalyze an S_N_2-reaction-mediated 2′-O methyl transfer [40], [41], [42]. Structure-based alignment of nsp16 with VP39, NS5 MTase and FtsJ highlights these four strictly conserved residues (Lys-46, Asp-130, Lys-170 and Glu-203) in nsp16 (Figure S2). In the crystal structure, the SAM methyl group stretches out to the surface provided by the K-D-K-E motif (Figures 7B and S3), which are located at the bottom of the central groove, and might bind the first adenine nucleotide, conserved in SARS genomic and subgenomic RNAs, as the acceptor of methyl group during the methylation. This was also demonstrated by the crystal structures of other 2′-O MTases [40], [43], [44], [45]. The central groove of nsp16 is positively charged (Figures 7C and 7D), and the phosphate backbone of the cap-containing RNA is highly negatively charged. These observations indicate that the central groove in nsp16 is most likely the cap binding site. However, the cap binding groove of nsp16 is built by two flexible loops (residues 26-38 and residues 130–148) in nsp16 (Figures 7A and 7B), which replace the highly stable α-helices (A1, A2 and half of the αD) in flavivirus NS5 MTase along the cap-binding groove [46] (Figure S1D). Therefore, it is obvious that the RNA binding groove of nsp16 is too flexible and unstable to hold the substrate cap-0 RNA (Figures 7A and 7B), which explains why nsp16 alone shows an extraordinary low affinity for both m7GpppA-RNA and m7GpppA cap analogue in biochemical assays (Figures 3A, lane 2, 3C and 3E).

To further characterize the substrate RNA binding site of nsp16/nsp10 complex, we performed molecular modeling of nsp16/nsp10 complex with m7GpppAAAAAA (m7GpppA-RNA) and m7GpppGAAAAA (m7GpppG-RNA), respectively (Figure 7C, 7D and 7E). m7GpppG-RNA was derived from the structure of vaccinia virus 2′-O-MTase VP39 (PDB entry: 1AV6), and m7GpppA-RNA was mutated manually from m7GpppG-RNA. As shown in Figures 7C and 7D, the first three transcriptional nucleotides are contacted with nsp16 and the following transcriptional nucleotides are contacted with nsp10. This docking model shows that nsp10 is involved in substrate RNA binding in nsp16/nsp10 complex, as the existence of nsp10 extends the positively charged area (Figures 7C and 7D), consequently elongating the RNA-binding groove of nsp16, which may increase the RNA-binding affinity.

In biochemical assays, nsp16/nsp10 complex indeed showed increased binding affinity for m7GpppA-capped RNA (Figures 3A and 3E) but still maintained a very low affinity for m7GpppA cap analogue (Figures 3C and 3E). These results indicate that m7GpppA alone is not long enough to reach the extended positively charged area provided by nsp10 and additional nucleotides following the m7GpppA cap are needed for binding to the nsp16/nsp10 complex. As shown previously, m7GpppAC_5_ acted as an effective substrate of nsp16/nsp10 complex [25], suggesting that as few as 5 extra nucleotides are sufficient. Nsp10 itself possesses two zinc-finger motifs and has the ability to bind polynucleotides nonspecifically in the presence of zinc ions (Figure 3A, lane 5) [37]; therefore the RNA binding by the nsp10 portion is not sequence-specific. In conclusion, the RNA-binding groove extension provided by nsp10 may contribute to hold the extended RNA chain following the m7GpppA cap and stabilize the interaction between m7GpppA-RNA and nsp16 cap binding site.

By analysis of RNA binding site of nsp16/nsp10 complex, an unexpected promontory composed of residues 74–77 at the catalysis activity surface of nsp16 could be readily identified, which might have steric hindrance for binding GpppG-capped RNA. The residue Asp-75 (D75) stretches out to the C2 atom of first transcribed nucleotide (Figure 7E) and may thus functions as the specificity determinant. We also performed structural alignment analysis between nsp16/nsp10 complex and VP39 (PDB entry: 1AV6) at the methyltransferase activity site (Figure 7F and 7G). Compared with VP39, the binding pocket for substrate RNA of nsp16/nsp10 complex appears more limited, due to the existence of residues 74–77, especially D75. The amino group connected to C2 atom of first transcribed guanylic acid in m7GpppG-RNA seems too close to the side chain of D75 compared with m7GpppA-RNA, which has no amino group at C2 atom. This structural difference might explain why m7GpppG-RNA exhibits a relatively low affinity to the nsp16/nsp10 complex (Figure 3E).

## Discussion

SARS-CoV nsp16 is the only 2′-O-MTase currently known that needs a stimulatory subunit for exerting its methyltransferase activity. In this work, we showed that nsp10 could stimulate nsp16 to bind the methyl donor SAM and the capped RNA substrate. These mechanisms could be explained based on the crystal structure of nsp16/nsp10 complex, and confirmed by mutational analysis. This is reminiscent of the activation mechanism of the N7-MTase involved in vaccinia virus mRNA capping [47]. The vaccinia N7-MTase consists of two subunits, the catalytic subunit located in the C-terminal domain of D1 protein and the stimulatory subunit D12. The activation of D1 MTase activity by D12 is achieved through increase of the substrate and co-substrate affinity as well as enhancement of the stability of D1 protein [47]. Structural analysis revealed that D12 is structurally homologous to cap 2′-O-MTase with a truncation of the SAM-binding domain [48]. In contrast, SARS-CoV nsp10 does not possess an MTase fold and is structurally not similar to any other proteins deposited in the PDB database [35], [36]. Vaccinia virus VP39 protein acts as the viral 2′-O-MTase but it does not require a stimulatory factor for its enzymatic activity [40], [49].

Based on the crystal structure of nsp16/nsp10 complex and biochemical analysis, the mechanisms of nsp16 binding to substrates m7GpppA-RNA and SAM with the assistance of nsp10 were revealed. Our data showed that nsp10 acts as a buttress supporting the seemingly flexible loop 100–108 critically involved in SAM-binding (Figures 6B and 6C) and thus enhancing the SAM-binding affinity. For binding of nsp16 to capped RNA, it appears in the structure that the RNA binding groove in nsp16 has only sufficient space for binding the 5′-cap of the RNA, but for stable interaction of nsp16 and capped RNA substrate, nsp10 is needed to extend the groove and accommodate extra nucleotides following the cap (Figures 3 and 7). The residues Lys-93 and His-80 in nsp10 are involved in the interaction with the loop 100–108 of nsp16, and our results showed that the K93A mutation reduced the SAM-binding activity significantly but not the RNA-binding affinity (Figures 5C and 5D), indicating that this site is essential for SAM-binding but not for the overall interactions of nsp16 and nsp10 [39]. Similar results were obtained for H80R mutation (data not shown). The K93A mutation caused an overall decrease by 60% in the 2′-O MTase activity of nsp16/nsp10 complex (Figures 5A and 5B). The mutational and biochemical analysis of this and previous studies [39] further proved the structural model for the SAM and RNA binding mechanisms. It was reported that alanine replacements of nsp10 in murine hepatitis virus (MHV) resulting in lethal phenotypes mapped to a central core of nsp10 that is resistant to mutation, and the rescued viruses with mutations in nsp10 reduced viral RNA synthesis [50]. As most of these mutations of nsp10 were located at the interaction interface of nsp16/nsp10 complex, they might influence the activities of viral 2′-O-MTase.

The crystal structure of nsp10 in nsp16/nsp10 complex is the same as the nsp10 monomer [35]. Compared the crystal structure of nsp16/nsp10 complex with dodecamer of nsp10 [36], we found that the interaction surface of nsp16-nsp10 has partial overlap with the contact surface of nsp10 within dodecamer. Also the surface of nsp10 which may associate with nsp16 faces to the inner space of spherical nsp10 dodecamer, which does not leave sufficient space for nsp16 binding. This indicates that nsp10 dodecamer structure may not be involved in the stimulation of nsp16 2′-O-MTase activity. However, the existence of nsp10 dodecamer structure during viral infection could not be excluded because nsp10 is translated up to several times more than nsp16 [51], [52] and the surplus nsp10 may form structures other than the nsp16/nsp10 complex. In addition, nsp10 is found in the nsp4-nsp10 precursor [53] and abolishment of the nsp9-nsp10 cleavage site resulted in viable virus [54]. This observation suggests that fusion of nsp10 with nsp9 should not disrupt the essential activities of nsp10 involved in virus replication. In the nsp16/nsp10 complex, the N-terminal part of nsp10, which connects with nsp9 in the nsp9-nsp10 fusion, is exposed at the surface of the complex. The first 9 amino acids at the very N-terminus of nsp10 are invisible in the crystal structure, indicating that they might form a flexible loop. In addition, the C-terminus of nsp9 is located at the surface of this non-specific single-stranded RNA binding protein [51], [55]. Taken together, this suggests that the nsp9-nsp10 is structurally capable of forming a complex with nsp16 and may thus stimulate the 2′-O-MTase activity of nsp16. The nsp9-nsp10 fusion has less propensity to form a dodecamer, which may further weaken the biological relevance of the nsp10 dodecamer structure observed previously [36].

Nsp10 represents a multi-functional protein involved in viral RNA synthesis, polyprotein processing and RTC assembly [50], [53], [54]. It was shown that nsp10 could interact with both 2′-O-MTase nsp16 and N7-MTase nsp14 [30], [31], suggesting that a single nsp10 molecule or its dimer could associate with both nsp16 and nsp14 at the same time in the RTC. Thus, one model can be proposed to explain the RNA cap methylation during coronavirus replication: After translation and processing of polyproteins 1a and 1ab, the mature nsp14, nsp16 and nsp10 form the RNA methylation apparatus, where SAM is bound by nsp14 and nsp16 in the presence of nsp10. The newly transcribed viral RNA is capped by the unknown capping enzyme (GTase) and bound to nsp10. The 5′-end of the RNA is first associated with nsp14 and methylated at the N7 position of the cap guanine. Next, the conformation of the RNA-protein complex is altered, and the 5′-end of viral RNA is transferred from the RNA-binding groove of nsp14 to that of nsp16, resulting in second methylation at the 2′-O-site in the ribose of the first nucleotide following the cap. Further experiments are needed to confirm this model.

Cellular and DNA virus capping enzymes generally are not sequence-specific as they accommodate a large number of different mRNA species. However, the genomes of RNA viruses are very small in comparison with DNA genomes and usually encode just a few genomic and subgenomic mRNAs with conserved 5′-ends. It has been shown that the flavivirus MTases are sequence-dependent [42], [56]. In our previous work, we showed that the SARS-CoV nsp14 N7-MTase is sequence-unspecific as it could methylate the RNA cap of different RNAs both in vitro and in yeast cells [8]. However, in the current study, we showed that the nsp16 2′-O-MTase is sequence-dependent as it could only methylate m7GpppA-capped RNA, where the first nucleotide is absolutely restricted to adenosine. Structural modeling analysis suggests that the amino residues at positions 74–77 of nsp16 may be the determinant for such sequence specificity (Figures 7C, 7D and 7E). In addition, the unknown coronavirus GTase may also contribute to the specificity of coronavirus capping enzymes. In coronavirus life cycle, genomic RNA replication and subgenomic RNA transcription take place in association with double-membrane vesicle [3], and this physical restriction may make the capping apparatus accessible only to viral RNAs.

It is well known that mRNA capping and methylation play important roles in mRNA stability, processing, transport and protein translation. Very recently, it was found that 2′-O-methylation of the viral RNA cap is essential for RNA viruses to avoid innate immune recognition by the host immune system [23], [24]. Thus, inhibition of viral MTase activity should be able to suppress viral replication and attenuate viral virulence in infection and pathogenesis. The MTase active site has been suggested as a drug target for developing antiviral drugs [57], [58], [59]. However, the MTase fold is structurally conserved between viral and cellular MTases, and it is thus difficult to obtain antiviral compounds with high specificity targeting MTase active sites. For this reason, it looks more promising to target the interface of nsp16 and nsp10, which is unique to coronaviruses.

In summary, we have characterized the SARS-CoV 2′-O-MTase and the activation mechanism of nsp16 by nsp10 biochemically and structurally. We found that nsp10 promoted the substrate and co-substrate binding of nsp16 by increasing the stability of the SAM-binding pocket and by extending the RNA-binding groove of nsp16. The current findings not only provide insights into the mechanism of SARS-CoV 2′-O-methylation but also facilitate design and development of highly specific antiviral drugs targeting the nsp16/nsp10 interface.

## Materials and Methods

### Protein expression and purification

The coding sequences of SARS-CoV nsp10-nsp11 fusion protein and nsp16 were PCR amplified from the cDNA sequence of SARS-CoV strain WHU (gi: 40795428) [2], [31] using the primers as listed in Table S1. The nsp10-nsp11 fusion protein and nsp16 genes (encoding residues Asn4240-Val4382 [35] and Ala6776-Asn7073 of replicase pp1ab) were cloned into pET30a (Novagen) (pET30a-His_6_-nsp10-nsp11, pET30a-His_6_-thrombin-nsp16) to produce recombinant proteins carrying an N-terminal His_6_-tag. The mutants of nsp10 (G70A, K93A, Y76A/C77A/R78A, and H83A/P84A) were generated by overlap PCR with mutagenic primers (Table S1) and cloned into pET30a as described for wild-type nsp10-nsp11 fusion protein. All constructs were verified by DNA sequencing. Both pET30a-His_6_-nsp10-nsp11 and pET30a-His_6_-thrombin-nsp16 transformed *E. coli* BL21 (DE3) cells were grown at 37°C in Luria-Bertani (LB) medium with 50 µg/mL kanamycin and induced with 0.4 mM isopropyl β-D- thiogalactopyranoside (IPTG) at 16°C for 12–16 hours. The SARS-CoV unique domain (SUD) of nsp3 (nsp3-SUD), nsp5, N-terminal domain of nsp12 (nsp12N), and nsp14 protein expression and purification were described previously [8]. The sequences of the cDNA and proteins have been deposited in GenBank database with accession numbers listed at the end of “Materials and Methods” section.

To obtain nsp10-nsp11 and nsp16 protein complex, 1 L of pET30a-His_6_-nsp10-nsp11 cells and 2 L of pET30a-His_6_-thrombin-nsp16 cells were mixed together and resuspended in buffer A [50 mM Tris-HCl, pH 7.5, 150 mM NaCl, 5 mM MgSO_4_, 5% glycerol] supplemented with 10 mM imidazole. After sonication and centrifugation, cleared lysates were applied to nickel-nitrilotriacetic acid (Ni-NTA) resin and washed with buffer A supplemented with a stepwise imidazole gradient of 20 mM, 50 mM, and 80 mM. Proteins were eluted with buffer A supplemented with 250 mM imidazole, and 0.5 mM SAM. After shaking at 4°C for 10 hours and centrifugation, the protein sample was further purified on a Superdex 200 column (GE) equilibrated with buffer A. Fractions containing the nsp16/nsp10 complex (nsp11 region was degraded during the process, data not shown) were concentrated to 10 mg/ml by ultrafiltration and frozen at −80°C for further use. The expression and purification conditions of selenomethionine (SeMet)-labeled nsp16 and nsp10 (unlabeled) protein complex were the same as for native nsp16/nsp10 complex except that modified M9 medium was used instead of LB medium during the expression of nsp16.

### Crystallization and data collection

Crystals were grown by the hanging-drop vapor diffusion method. The drops contained 1 µl each of nsp16/nsp10 protein complex [10 mg/ml in 50 mM Tris-HCl (pH 7.5), 150 mM NaCl, 5 mM MgSO_4_, 5% glycerol, 5 mM SAM] and 1 µL mother liquor. Protein crystals were obtained at 25°C after 24 h in 0.1 M MES, pH 5.0, 2 M NaCl, 0.1 M NaH_2_PO_4_, 0.1 M KH_2_PO_4_. The crystals of SeMet labeled nsp16/nsp10 complex were obtained in the same conditions. For data collection, the crystals were cryocooled (by nitrogen gas stream, 100 K) in the original mother liquor containing 20% (vol/vol) glycerol and diffraction data sets were collected on beamline BL-17U1 at Shanghai Synchrotron Radiation Facility (SSRF) for native protein complex crystal and beamline 1W2B at Beijing Synchrotron Radiation Facility (BSRF) for SeMet labeled nsp16/nsp10 complex crystal. The diffraction data were processed and scaled with the HKL2000 package. Data collection statistics are listed in Table S2.

### Structure determination and refinement

The structure was solved by the multi-wavelength anomalous diffraction (MAD) method based on two sets of derivative data and one set of native data. A preliminary model was readily built up by Phenix package [60] from derivative data, which covered around 60% of whole structure. Most of the secondary structures were obvious in the initial model, especially the 7-β-strand core. The model was then used for refining and manual building against high resolution native diffraction data. To speed up the refining process, the sole nsp10 structure was introduced into the complex by molecular replacement using Phaser [61] in CCP4 package [62]. The CNS suite [63] and Phenix refinement program (phenix.refine) [64] were used iteratively in refinement. Simulated annealing, position refining and B-factor refining were used in multiple rounds. The density for loop regions became visible gradually as refinement proceeded. A SAM molecule and coordinated zinc ion in nsp10 were included based on 2Fo-Fc electron density. Structure validation was performed periodically during refinement by Procheck [65], [66]. Eventually most of the protein sequences, except the disordered C-terminal tail in nsp10 and artificial tags generated from vectors, were involved in the final structure, and ordered water molecules were added.

### Molecular modeling

The methyltransferase domain in the structure of VP39 was superimposed to nsp16 in the nsp16/nsp10 complex by LSQKAB [67] in CCP4 package [62], and the RNA molecule in VP39 was used to model the interaction between RNA and SARS nsp16/nsp10 complex. The interface between RNA and nsp16/nsp10 was optimized by energy minimization using PHENIX [64] for 3 cycles.

### Preparation of RNA substrates

The ATP-initiated RNA substrates representing the 5′-terminal 259 nucleotides of the SARS-CoV genome and nonviral RNA substrate comprising 52 nucleotides (with G as the first nucleotide) were in vitro transcribed, ^32^P-labeled at cap structures (m7G*pppA-RNA, G*pppA-RNA, or m7G*pppG-RNA, where the * indicates that the following phosphate was radio-labeled.), and purified as previously described [8]. RNAs containing ^32^P-labeled cap-1 structure (m7G*pppAm-RNA) as positive control were converted from cap-0 structure m7G*pppA-RNA by a vaccinia virus 2′-O-methyltransferase VP39 following the manufacturer's protocol (Epicentre). RNAs containing unlabeled cap structures (m7GpppA-RNA or m7GpppG-RNA) were prepared by a vaccinia virus capping enzyme following the manufacturer's protocol (Epicentre) as well as ^3^H-labeled cap structures (*m7GpppA-RNA or *m7GpppG-RNA), except that 10 µCi of S-adenosyl [methyl-^3^H] methionine (67.3 Ci/mmol, 0.5 µCi/ul) was used as the methyl donor instead of cold SAM. The ^32^P-labeled cap analogue (m7G*pppA or m7G*pppG) and ^3^H-labeled cap analogue (*m7GpppA or *m7GpppG) were digested from m7G*pppA-RNA/m7G*pppG-RNA and *m7GpppA-RNA/*m7GpppG-RNA by nuclease P1 (Sigma) in 10 mM Tris-HCl, pH 7.5, 1 mM ZnCl_2_ at 50°C for 30 min. All the RNA substrates were extracted with phenol-chloroform and precipitated with ethanol. The unlabeled cap analogues (m7GpppA, GpppA, and m7GpppG) were purchased from New England BioLabs.

### Biochemical assays for MTase activity

Purified recombinant or mutant proteins (0.5 µg) and 2×10^3^ cpm of ^32^P-labeled m7G*pppA-RNA or G*pppA-RNA substrates were added to 8.5 µL reaction mixture [40 mM Tris-HCl (pH 7.5 or 8.0), 2 mM MgCl_2_, 2 mM DTT, 10 units RNase inhibitor, 0.2 mM SAM] and incubated at 37°C for 1.5 h. RNA cap structures were liberated with 5 µg of nuclease P1 (Sigma), then spotted onto polyethyleneimine cellulose-F plates (Merck) for TLC, and developed in 0.4 M ammonium sulfate. The extent of ^32^P-labeled cap was determined by scanning the chromatogram with a PhosphorImager [8].

MTase activity assays were carried out in 30 µL reaction mixture [40 mM Tris-HCl (pH 7.5), 2 mM MgCl_2_, 2 mM DTT, 40 units RNase inhibitor, 0.01 mM SAM], with 1 µCi of S-adenosyl [methyl-^3^H] methionine (67.3 Ci/mmol, 0.5 µCi/µl), 1 µg of purified proteins or mutant proteins, and 3 µg of m7GpppA/m7GpppG-RNA substrates or other RNA substrates (2 mM m7GpppA/GpppA/m7GpppG cap analogue or 15 mM NTPs) at 37°C for 1.5 h. ^3^H-labeled product was isolated in small DEAE-Sephadex columns and quantitated by liquid scintillation [68].

### SAM binding assay

25 µL reaction mixtures [40 mM Tris-HCl (pH 7.5), 2 mM MgCl_2_, 2 mM DTT] containing 1 µg of purified proteins and 1 µCi of *S*-adenosyl [methyl-^3^H] methionine (67.3 Ci/mmol, 0.5 µCi/µl) were pipetted into wells of a microtiter plate. The reaction mixtures were incubated on ice and irradiated with 254-nm UV light in a Hoefer UVC500 cross-linking oven for 30 min. The distance of samples from the UV tubes was 4 cm. The samples were then analyzed by 12% sodium dodecyl sulfate-polyacrylamide gel electrophoresis (SDS-PAGE). The gels were soaked in Enlightning Solution (PerkinElmer) and used for fluorography [68].

### Gel shift assay

The gel shift assay provides a simple and rapid method for detecting RNA-binding proteins. This method has been widely used in the study of sequence-specific RNA-binding proteins. In this assay, 1 µg of purified proteins and 4×10^3^ cpm of ^32^P-labeled RNA substrates (m7G*pppA-RNA, m7G*pppG-RNA, m7G*pppA cap analogue, and m7G*pppG cap analogue) were added to 20 µL reaction mixtures [40 mM Tris-HCl (pH 7.5), 2 mM MgCl_2_, 2 mM DTT]. The reactions were incubated at room temperature for 25 min, and separated by nondenaturing polyacrylamide gel (N-PAGE). The RNA substrate bands were quantitated by scanning the gels with a PhosphorImager.

### RNA binding assay

In each set of RNA binding assay, 3 µg of freshly prepared ^3^H-labeled RNA substrates (*m7GpppA-RNA, *m7GpppG-RNA, *m7GpppA cap analogue, and *m7GpppG cap analogue) and 4 µg of purified His_6_-proteins were mixed in 100 µL binding buffer [40 mM Tris-HCl (pH 7.5), 2 mM MgCl_2_]. The binding reactions were shaken at 4°C over night. 20 µL high affinity Ni-NTA resin (50% slurry, GenScript), equilibrated with binding buffer, were added to binding reactions and mixed gently for 30 min at 4°C. The complex of ^3^H-labeled-RNA-His_6_ and Ni-NTA-bound protein was pelleted by centrifugation for 20 s at 1000 g, and washed twice with binding buffer to remove free ^3^H-labeled RNA substrates. The complex was finally resuspended in 100 µL of binding buffer, and a 30 µL aliquot was analyzed by Western Blotting analysis with anti-His-tag antibody. The remaining 70 µL was quantitated by liquid scintillation.

### Isothermal Titration Calorimetry

ITC experiments on the interaction of SAM with nsp16, nsp10, and nsp16/nsp10 complex of SARS-CoV in the absence of DTT were carried out at 25°C using a VP-ITC titration calorimeter (MicroCal, Northampton, MA). Freshly purified nsp16 and nsp10 proteins were mixed, and their final concentrations were 10 µM and 80 µM, respectively. Then nsp16, nsp10, and nsp16/nsp10 complex were dialyzed against 40 mM Tris-HCl buffer (pH 8.0) containing 50 mM NaCl, over night at 4°C, extensively to remove glycerol and DTT. A solution of nsp16, nsp10, or nsp16/nsp10 complex was loaded into the sample cell (1.43 mL), and a solution of 150 µM SAM was placed in the injection syringe (300 µL). The first injection (5 µL) was followed by 29 injections of 10 µL. Dilution heats of SAM were measured by injecting SAM solution into buffer alone and were subtracted from the experimental curves prior to data analysis.

The interaction of nsp16, nsp10, and nsp16/nsp10 complex of SARS-CoV with m7GpppA-capped RNA in the absence of DTT were carried out at 25°C using an ITC200 titration calorimeter (MicroCal, Northampton, MA), which has higher sensitivity than the equipment adopted for analyzing protein-SAM binding as described above. A solution of m7GpppA-capped RNA (7 µM) was loaded into the sample cell (500 µL), and a solution of 100 µM purified proteins were placed in the injection syringe (40 µL). Dilution heats of purified proteins were measured by injecting purified proteins solution into buffer alone and were subtracted from the experimental curves prior to data analysis.

The resulting data were fitted to a single set of identical sites model using MicroCal ORIGIN software supplied with the instrument, and the binding stoichiometry, *N*, the standard molar enthalpy change for the binding, 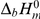, and the dissociation constant, *K*_d_, were thus obtained. The standard molar free energy change, 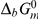, and the standard molar entropy change, 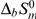, for the binding reaction were calculated by the fundamental equations of thermodynamics: 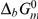 = 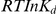; 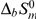 = (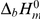-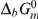)/T.

### Accession numbers

The GenBank accession numbers for genes and proteins mentioned in the text are as follow: SARS coronavirus nsp3 unique domain (SUD), JN247391; SARS coronavirus nonstructural protein nsp5, JN247392; SARS coronavirus nonstructural protein nsp10, JN247393; SARS coronavirus nsp10-nsp11 fusion protein, JN247394; SARS coronavirus nsp12 N-terminal domain (nsp12N), JN247395; SARS coronavirus nonstructural protein nsp14, JN247396; SARS coronavirus nonstructural protein nsp16, JN247397.

## Supporting Information

Figure S1 **Comparison of the surface features between nsp16/nsp10 and other 2′-O MTases.** (A) The SAM binding cleft of nsp16 built by three loop regions. Nsp10 is show as ribbon and colored in green. Nsp16 is shown as surface and colored in cyan. Loop 71–79, loop 100–108 and loop 130–148 regions are colored in yellow. SAM is shown as sticks and colored in magenta. Comparison of the surfaces of nsp16/nsp10 (B), vaccinia virus VP39 (PDB entry 1AV6) (C), Dengue virus NS5 MTase (PDB entry 1L9K) (D), *Escherichia coli* FtsJ (PDB entry 1EJ0) (E) and Bluetongue virus VP4 2′-O-MTase (PDB entry 2JHP) (F). Proteins are shown as surface and colored by secondary structure (α helix: red, β strand: yellow, loop: green). SAM is shown as sticks and colored by atoms (C: salmon, O: red, N: blue, H: white).
(TIF)

Figure S2 **Structure-based sequence alignments of nsp16 and nsp10.** (A) Sequence alignment of representative mRNA cap 2′-O-MTases from vaccinia virus VP39 (PDB entry 1VPT), Flavivirus NS5 MTase (PDB entry 1L9K), and FtsJ (PDB entry 1EIZ) with nsp16 of SARS-CoV. The secondary structure of VP39 is shown above and that of nsp16 below the alignment. Residues with 100% conservation are indicated in solid red boxes and those with identity of 70% or higher are depicted in light red color. Red arrowheads indicate the conserved K-D-K-E motif in 2′-O-MTases. The short section of nsp16 αD helix as compared with other 2′-O-MTases and the flexible loop 130–148 of nsp16 are underlined in yellow. (B) Sequence and secondary structure of coronavirus nsp10 from SARS-CoV, infectious bronchitis virus (IBV), human coronavirus OC43 (HCoV-OC43), bovine coronavirus (BcoV), murine hepatitis virus (MHV), transmissible gastroenteritis virus (TGEV). The secondary structure of SARS-CoV nsp10 is shown above.
(TIF)

Figure S3 **K-D-K-E surface site in the central groove of nsp16.** Nsp16 is shown as surface with 20% transparency (A) and ribbon (B). Lys-46, Asp-130, Lys-170 and Glu-203 are shown as sticks and colored in magenta. Nsp10 is shown as ribbon and colored in green. SAM is shown as sticks and colored in yellow. The methyl group of SAM is colored in blue. Zinc ions are shown as spheres.
(TIF)

Table S1 **Primers used for PCR cloning of SARS-CoV gene fragments into E. coli expression vector pET30a.**
(DOC)

Table S2 **X-ray crystallographic data and refinement statistics for nsp16/nsp10/SAM complex.**
(DOC)
