# Supplementary figures and images for: Biochemical and Structural Insights into the Mechanisms of SARS Coronavirus RNA Ribose 2′-O-Methylation by nsp16/nsp10 Protein Complex
Source: PLoS Pathog. 2011 Oct 13;7(10):e1002294. doi: 10.1371/journal.ppat.1002294 (PMC3192843; doi:10.1371/journal.ppat.1002294)

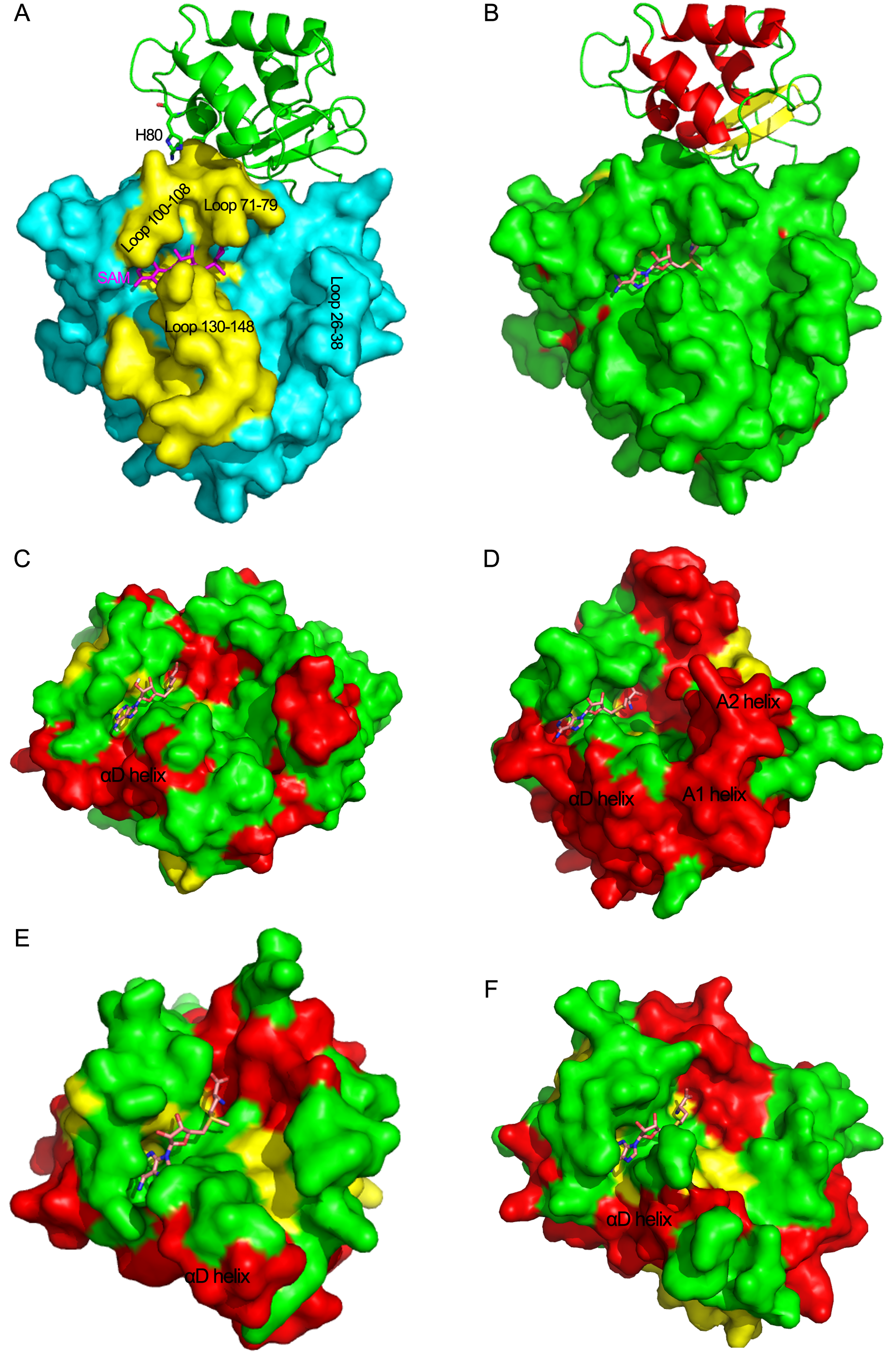

Supplement: Figure S1 — Comparison of the surface features between nsp16/nsp10 and other 2′-O MTases. (A) The SAM binding cleft of nsp16 built by three loop regions. Nsp10 is show as ribbon and colored in green. Nsp16 is shown as surface and colored in cyan. Loop 71–79, loop 100–108 and loop 130–148 regions are colored in yellow. SAM is shown as sticks and colored in magenta. Comparison of the surfaces of nsp16/nsp10 (B), vaccinia virus VP39 (PDB entry 1AV6) (C), Dengue virus NS5 MTase (PDB entry 1L9K) (D), Escherichia coli FtsJ (PDB entry 1EJ0) (E) and Bluetongue virus VP4 2′-O-MTase (PDB entry 2JHP) (F). Proteins are shown as surface and colored by secondary structure (α helix: red, β strand: yellow, loop: green). SAM is shown as sticks and colored by atoms (C: salmon, O: red, N: blue, H: white). (TIF) [file ppat.1002294.s001.tif]

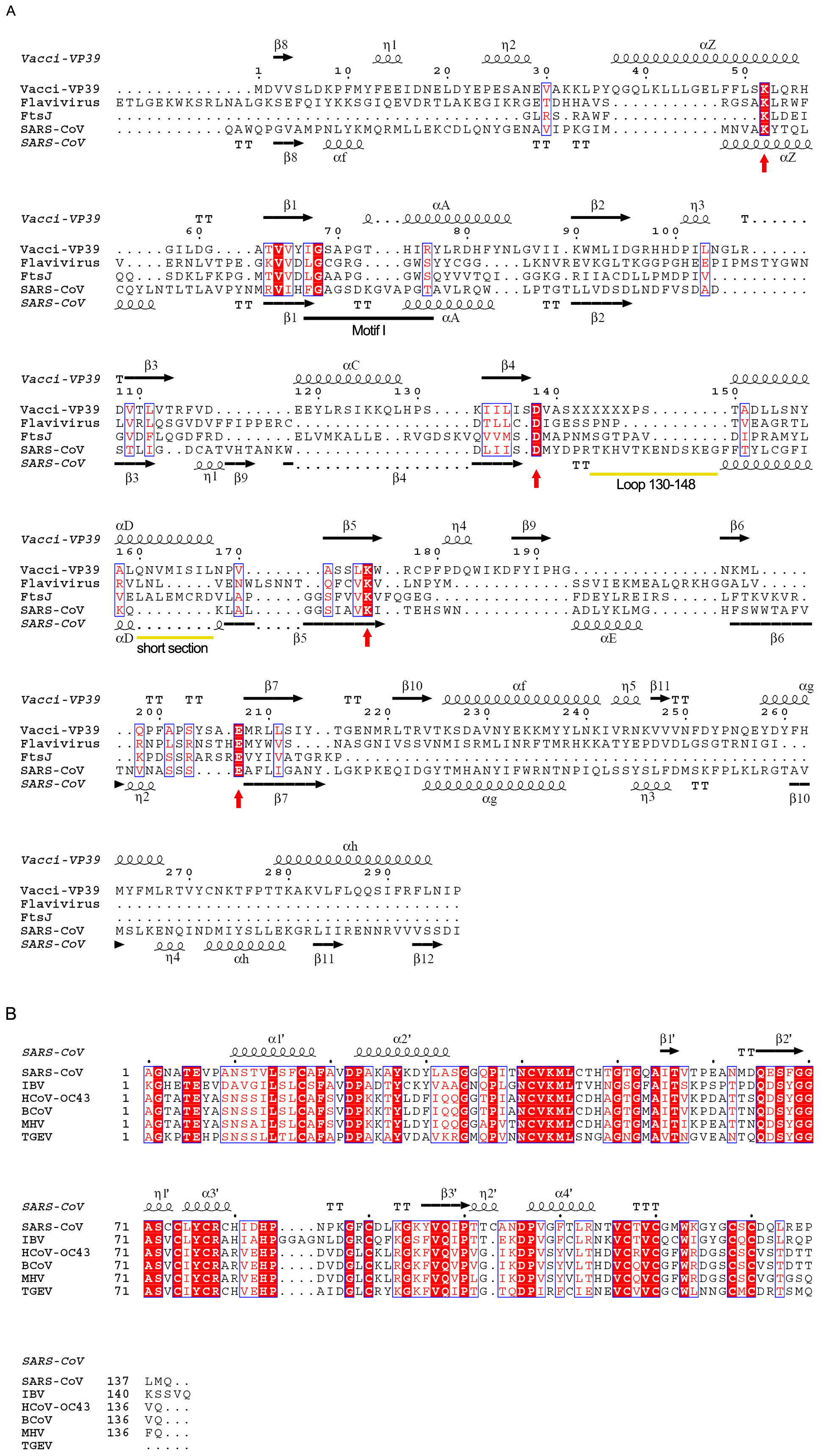

Supplement: Figure S2 — Structure-based sequence alignments of nsp16 and nsp10. (A) Sequence alignment of representative mRNA cap 2′-O-MTases from vaccinia virus VP39 (PDB entry 1VPT), Flavivirus NS5 MTase (PDB entry 1L9K), and FtsJ (PDB entry 1EIZ) with nsp16 of SARS-CoV. The secondary structure of VP39 is shown above and that of nsp16 below the alignment. Residues with 100% conservation are indicated in solid red boxes and those with identity of 70% or higher are depicted in light red color. Red arrowheads indicate the conserved K-D-K-E motif in 2′-O-MTases. The short section of nsp16 αD helix as compared with other 2′-O-MTases and the flexible loop 130–148 of nsp16 are underlined in yellow. (B) Sequence and secondary structure of coronavirus nsp10 from SARS-CoV, infectious bronchitis virus (IBV), human coronavirus OC43 (HCoV-OC43), bovine coronavirus (BcoV), murine hepatitis virus (MHV), transmissible gastroenteritis virus (TGEV). The secondary structure of SARS-CoV nsp10 is shown above. (TIF) [file ppat.1002294.s002.tif]

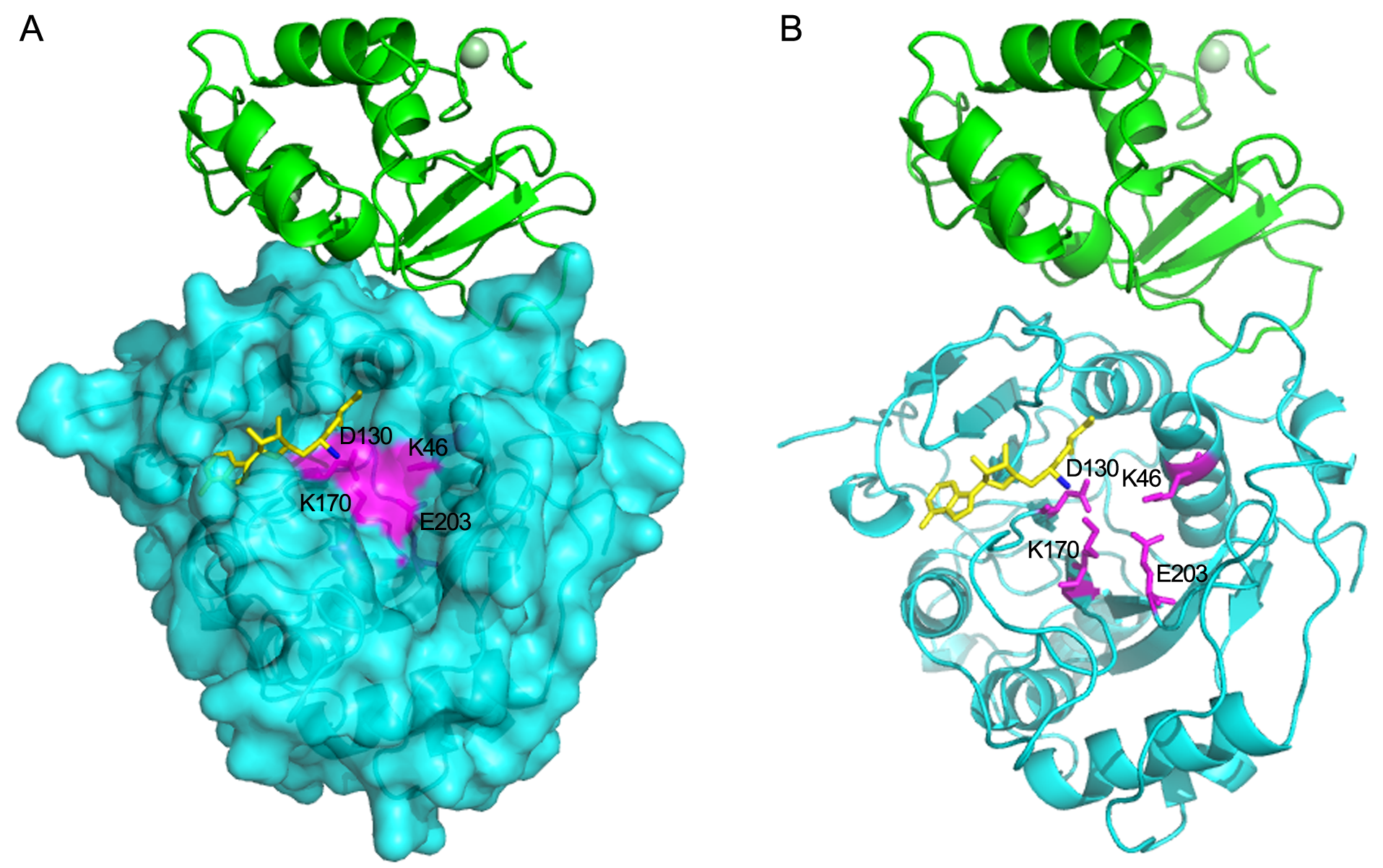

Supplement: Figure S3 — K-D-K-E surface site in the central groove of nsp16. Nsp16 is shown as surface with 20% transparency (A) and ribbon (B). Lys-46, Asp-130, Lys-170 and Glu-203 are shown as sticks and colored in magenta. Nsp10 is shown as ribbon and colored in green. SAM is shown as sticks and colored in yellow. The methyl group of SAM is colored in blue. Zinc ions are shown as spheres. (TIF) [file ppat.1002294.s003.tif]
