# Supplementary material for: Biochemical and Structural Insights into the Mechanisms of SARS Coronavirus RNA Ribose 2′-O-Methylation by nsp16/nsp10 Protein Complex
Source: PLoS Pathog. 2011 Oct 13;7(10):e1002294. doi: 10.1371/journal.ppat.1002294 (PMC3192843; doi:10.1371/journal.ppat.1002294)
Supplement: Table S1 — Primers used for PCR cloning of SARS-CoV gene fragments into E. coli expression vector pET30a. (DOC) [file ppat.1002294.s004.doc]

Table S1. Primers used for PCR cloning of SARS-CoV gene fragments into *E. coli* expression vector pET30a.

| **Primer** | **Sequence (5'→3')** | **Position** |
| --- | --- | --- |
| SARS-nsp10-nsp11-F | TGCCATATGGGATCTGATAAAATTCATCATCATCATCATCACAATTCAACTGTGCTTTC | 12982-13413^*^ |
| SARS-nsp10-nsp11-R | CCGCTCGAGTTATTACACCGCAAACCCGTTTAAAAAC |
| SARS-nsp16-F | GCCCATGGCTGCAAGTCAAGCGTGG | 20589- 21482^*^ |
| SARS-nsp16-R | GCCTCGAGTCAGTTGTTAACAAGAATATCAC |
| nsp10^†^ G70A-F | TTGGTGCTGCTTCATGTTGTCTGTATTGTAGATGCCA | 13157- 13193^*^ |
| nsp10 K93A-F | ACTTGGCAGGTAAGTACGTCCAAATACCTACCA | 13226- 13258^*^ |
| nsp10 Y76A/C77A/R78A-F | TGTCTGGCTGCTGCATGCCACATTGACCATCCAAATC | 13174-13210^*^ |
| nsp10 H83A/P84A-F | ATTGACGCTGCAAATCCTAAAGGATTCTGTGACTTG | 13195- 13230^*^ |

F, Forward primer. R, Reverse primer. nsp, indicates non-structural protein.

*Numbering refers to the nucleotide coordinates of the SARS-CoV isolate WHU (GenBank Accession No.AY394850).

†Nsp10 refers to SARS-CoV non-structural protein nsp10.
