# Supplementary material for: Biochemical and Structural Insights into the Mechanisms of SARS Coronavirus RNA Ribose 2′-O-Methylation by nsp16/nsp10 Protein Complex
Source: PLoS Pathog. 2011 Oct 13;7(10):e1002294. doi: 10.1371/journal.ppat.1002294 (PMC3192843; doi:10.1371/journal.ppat.1002294)
Supplement: Table S2 — X-ray crystallographic data and refinement statistics for nsp16/nsp10/SAM complex. (DOC) [file ppat.1002294.s005.doc]

Table S2. X-ray crystallographic data and refinement statistics for nsp16/nsp10/SAM complex.

| **Crystals** | Native | Selenomethionine-derivative (peak ) | Selenomethionine-derivative (inflection) |
| --- | --- | --- | --- |
| **Data collection** |  |  |  |
| Space group | C222_1_ | C222_1_ | C222_1_ |
| Wavelength (Å) | 0.9793 | 0.9793 | 0.9795 |
| Unit cell dimensions |  |  |  |
| a (Å) | 67.33 | 67.38 | 67.39 |
| b (Å) | 184.52 | 184.36 | 184.38 |
| c (Å) | 128.59 | 128.56 | 128.59 |
| α, β, γ (º) | 90, 90, 90 | 90, 90, 90 | 90, 90, 90 |
| Molecules per ASU^Ф^ | 1 | 1 | 1 |
| Resolution (Å) ^*^ | 2.0(2.03-2.00) | 2.8(2.87-2.80) | 3.0(3.05-3.00) |
| Completeness (%)^*^ | 99.9 (99.2) | 99.4(98.7) | 98.8(96.3) |
| Redundancy^*^ | 6.2 (5.3) | 5.8 (5.0) | 5.3(4.4) |
| No. of total reflections | 335128 | 215655 | 185182 |
| No. of unique reflections | 54053 | 37182 | 34940 |
| I/σ^*^ | 13.9 (2.8) | 11.0 (2.4) | 10.2(1.9) |
| R_sym_^*†^ | 6.7 (31.5) | 7.9 (36.5) | 9.6(40.5) |
| Figure of merit |  | 0.54 | |
| **Refinement statistics** |  |  |  |
| Resolution (Å) | 2.0 |  |  |
| No. of reflections | 54053 |  |  |
| R_work_/R_free_ (%)^‡§^ | 19.3/22.2 |  |  |
| No. of atoms |  |  |  |
| Protein | 3205 |  |  |
| Ligand/ion | 29 |  |  |
| Water | 467 |  |  |
| B-factors (Å^2^) |  |  |  |
| Protein | 30.17 |  |  |
| Water | 39.64 |  |  |
| R.m.s. deviations |  |  |  |
| Bond length (Å) | 0.007 |  |  |
| Bond angle (º) | 1.165 |  |  |
| **Ramachandran analysis** |  |  |  |
| Most favored (%) | 88.6 |  |  |
| Additional allowed (%) | 9.7 |  |  |
| Generously allowed (%) | 1.7 |  |  |
| Disallowed (%) | 0.0 |  |  |

ФASU=asymmetric unit.

*Values in parentheses are for the highest resolution shell.

†Rsym = Σ|I-<I>|/Σ<I>, where I is the observed intensity, and <I> is the average intensity of multiple observations of symmetry related reflections.

‡R = Σhkl||Fobs|-|Fcalc||/Σhkl|Fobs|

§Rfree is calculated from 5% of the reflections excluded from refinement.
